# Coevolution Pattern and Functional Conservation or Divergence of miR167s and their targets across Diverse Plant Species

**DOI:** 10.1038/srep14611

**Published:** 2015-10-13

**Authors:** Suvakanta Barik, Ashutosh Kumar, Shabari Sarkar Das, Sandeep Yadav, Vibhav Gautam, Archita Singh, Sharmila Singh, Ananda K. Sarkar

**Affiliations:** 1 National Institute of Plant Genome Research, Aruna Asaf Ali Marg, New Delhi 110067; 2 International Centre for Genetic Engineering and Biotechnology (ICGEB), Aruna Asaf Ali Marg, New Delhi 110067, India

## Abstract

microRNAs (miRNAs), a class of endogenously produced small non-coding RNAs of 20–21 nt length, processed from precursor miRNAs, regulate many developmental processes by negatively regulating the target genes in both animals and plants. The coevolutionary pattern of a miRNA family and their targets underscores its functional conservation or diversification. The miR167 regulates various aspects of plant development in *Arabidopsis* by targeting *ARF6* and *ARF8.* The evolutionary conservation or divergence of miR167s and their target genes are poorly understood till now. Here we show the evolutionary relationship among 153 *MIR167* genes obtained from 33 diverse plant species. We found that out of the 153 of miR167 sequences retrieved from the “miRBase”, 27 have been annotated to be processed from the 3′ end, and have diverged distinctively from the other miR167s produced from 5′ end. Our analysis reveals that gma-miR167h/i and mdm-miR167a are processed from 3′ end and have evolved separately, diverged most resulting in novel targets other than their known ones, and thus led to functional diversification, especially in apple and soybean. We also show that mostly conserved miR167 sequences and their target *AUXIN RESPONSE FACTORS* (*ARF*s) have gone through parallel evolution leading to functional diversification among diverse plant species.

Endogenously produced small non-protein coding RNAs of 21–24 nucleotides (nts) have been extensively studied in recent years for their involvement in diverse biological processes in animal and plant development^1^. Changes in the critical sequences of a mature miRNA as well as its complementary sequence of the target genes may lead to functional diversification within or among species. In higher plants, two major classes of small regulatory RNAs, microRNAs (miRNAs) and trans-acting small interfering (ta-siRNAs) have been implicated in different aspects of plant development^2^. Some of the small RNAs are also regulated by plant hormones, nutrient availability and environmental stresses, which in turn affect developmental processes. The miRNAs, derived from a segment of the genome that is distinct from predicted protein coding regions, function as negative regulators of target genes mostly through the cleavage of target mRNAs^1^,^3^. The two conjugative action of *DICER LIKE1* (*DCL1*), with other associated factors, convert the primary transcripts of *MIRNA* genes (*pri-miRNAs*) to specific ~21 nt mature miRNAs through processing of concerned stem-loop structures of precursor miRNAs (pre-miRNAs)^1^,^4^,^5^,^6^,^7^. These mature miRNAs are then loaded into the RNA induced silencing complex (RISC)^8^,^9^ followed by pairing with target mRNAs^10^ to direct post transcriptional gene silencing (PTGS) or to inhibit translation of mRNAs^11^.

Plant miRNAs, which are the second most abundant small RNAs^12^, act as powerful endogenous regulators. The enriching reports on miRNAs in plant show that many miRNAs target transcripts encoding an array of transcription factors that control plant development and phase transition in *Arabidopsis*, maize and woody species^3^,^13^,^14^,^15^,^16^,^17^,^18^ while some others are involved in stress response and disease resistance^19^,^20^.

Plant miRNAs mostly regulate gene expression by binding to target mRNAs in a perfect or near-perfect complementary site^21^,^22^. This suggests that the miRNA-target modules could be conserved in long evolutionary time scales. Earlier studies based on experimental and computational analysis from *Arabidopsis* have indicated that many plant miRNAs and their targets are conserved between monocot and dicot plant groups^23^,^24^,^25^,^26^,^27^. Conserved miRNAs play an important role in conserved gene regulation such as regulation of leaf patterning, flower morphology and signal transduction, root nodulation^28^,^29^ etc. Plant hormone auxin (e. g. IAA, Indole-3-acetic acid) regulates various aspects of plant growth and development as well as response to environmental stress^30^,^31^. The auxin signaling is normally initiated or mediated through DNA binding proteins known as AUXIN RESPONSE FACTORs (ARFs) family^32^,^33^. The ARF proteins, possessing a conserved B-3 like DNA-binding domain, recognize auxin responsive *cis*-acting element (AuxRE) present in the promoter region of auxin-responsive genes to activate or repress their transcription^34^. Different ARF proteins have been implicated in embryogenesis, root development and floral organ development^35^,^36^. Among the *ARF* family members, *ARF6* and *ARF8*, which affect female and male fertility and adventitious root development in *Arabidopsis*, are negatively regulated by miR167, which cleave their mRNAs in the complementary regions. Over expression of miR167 as well as *arf6-2* and *arf8-3* mutants display floral defects and defect in ovule and anther development, whereas flowers expressing *ARF6/8* resistant to miR167 mediated cleavage are also sterile^37^,^38^,^39^,^40^,^41^.

Since precursor sequences of *MIRNA*s are much larger (than matures) and represent major part of the transcripts, the phylogenetic analysis with precursors is likely to reflect the true evolutionary history. However, similar to a critical domain of a conserved protein family, sequence variation in the mature miR167s, the functional region, is ultimately responsible for functional diversification, if any. Therefore, sequence comparison and phylogenetic analysis of mature miR167s should reflect their functional similarity and diversification in correlation with precursors across diverse plant species. Although the evolution of *ARF* genes have been previously studied^42^, the evolutionary changes in the miR167 binding sequences of its target *ARF6/ARF8*, which is crucial for balancing their abundance, has not been studied. Therefore, this is absolutely necessary to address the coevolution of miR167s and their target sequences (*ARF6/ARF8*/ other genes) to unravel the functional conservation or divergence of functionally important miR167s among diverse groups of plants. To understand the evolution of the miR167s, we analyzed one hundred fifty three mature and precursor miR167 sequences. From the sequence dataset, we reconstructed the evolutionary history of mature and precursor sequences of *MIR167* family members among thirty three diverse plant species, compared the phylogeny of miR167s to the previously studied evolutionary pattern for ARFs^43^, and uncovered the coevolutionary pattern of their known targets *ARF6* and *ARF8*.

## Results

The availability of well annotated complete genome sequences of diverse model land plants such as *P. patens, O. sativa A. thaliana* and *Z. mays* (as described in the materials & methods) have enabled the comparative genomics studies to explore the evolutionary relationship of the *pre-MIR167* gene family and their targets across diverse plant species. As miR167 is a crucial family of plant miRNA implicated in multiple biological processes including gametophyte development, flower development and adventitious root development, we have attempted to trace back the evolutionary relationship of miR167 family members (as registered in miRBase database registry) and their target sequences among the land plants.

### Identification of precursor and mature sequences of miR167s

**Figure 1:**
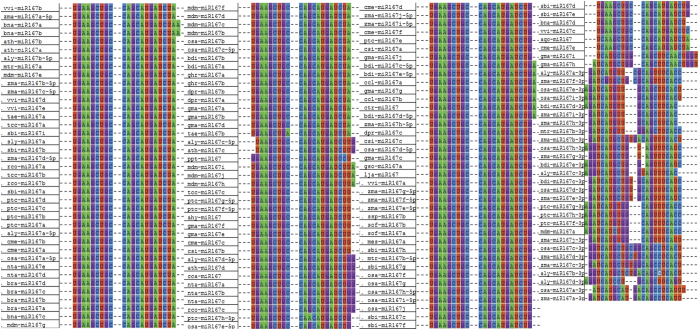
ClustalW alignment of one hundred and fifty three miR167 sequences retrieved from miRBase database registry (version 19) using MEGA5. Manual curation of aligned sequences produced fourteen unique sequences.

**Figure 2:**
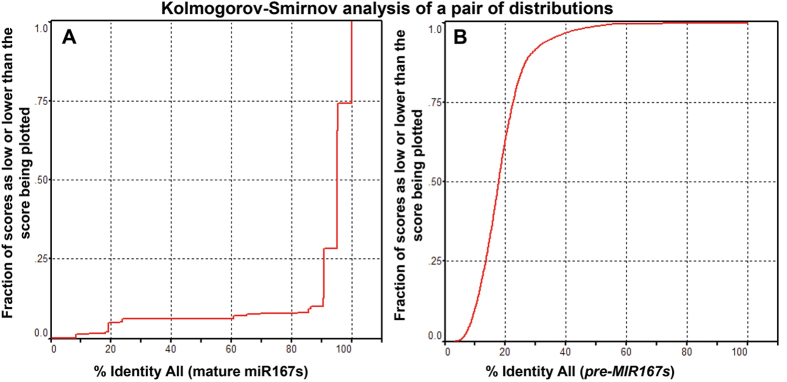
The percentage identity of the aligned miR167 sequences calculated using Kalmogorov-Smirnov statistical test in GeneDoc (version 2.7) sequence editing tool. (**A**) Percentage Identity of all one hundred and fifty three mature miR167 sequences. The test shows that ~0.25 fraction of sequences have ~90% sequence identity. Similarly ~0.25 fraction of the total sequences in *MIR167* (**B**) have >22% sequence identity.

We have identified 153 mature miR167 sequences from thirty three different plant species using miRBase Registry database (Table 1). The procedure of sequence identification has been explained in materials and methods section. Among these sequences, twenty seven sequences (number in each species is shown in parentheses) from six species namely *Arabidopsis lyrata* (4), *Brachypodium distachyon* (3), *Medicago truncatula* (1), *Oryza sativa* (6), *Populus trichocarpa* (3) and *Zea mays* (10) were found to be processed from 3′ end of the stem-loop sequences (Table 1). Apart from these, three other sequences are processed from 3′ end of stem loop sequences of gma-miR167h, gma-miR167i and mdm-miR167a, which we have observed in our analysis using the Mfold^44^ and RNAshape software tools. Unlike the miR166 sequences, where sequences were intermingled in Multiple Sequence Alignment (MSA)^45^, the miR167 sequences from different species (as specified in Table 1) taken for our studies are aligned at a distinct position (Fig. 1). Percentage Identity of aligned sequences, using Kalmogorov-Smirnov statistical test in GeneDoc (version 2.7), shows that ~0.25 fraction of mature miR167 sequences have ~90% sequence identity. Similarly, ~0.25 fraction of the total/precursor sequences (*pre-MIR167)* have >22% sequence identity (Fig. 2). This indicates that mature miR167s are more conserved than their precursors or entire genes.

### Phylogenetic analysis of mature miR167 sequences

**Figure 3:**
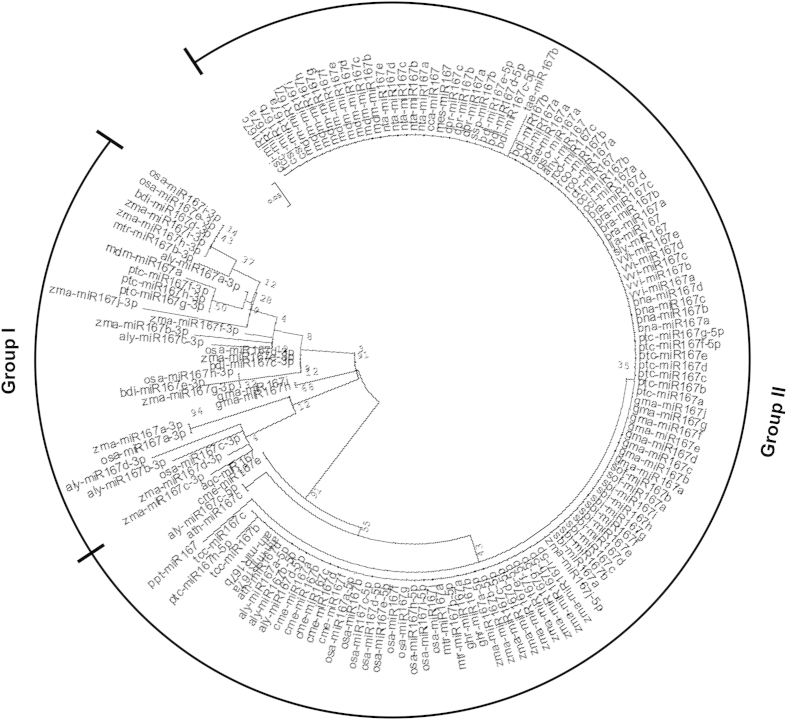
An unrooted ML phylogeny of miR167s using MEGA5. The tree is divided into two groups such as group I and group II. The group I contains three miR167-5p (processed from 5′ of precursor) sequences such as gma-miR167h/i and mdm-miR167a along with all miR167-3ps. All other miRNA167s (−5p) belong to group II. The scale bar represents the nucleotide substitution rate.

For the phylogenetic based comparative evolutionary study, we used Maximum Likelihood (ML) as well as Neighbor Joining (NJ) methods with the above mentioned parameters. The topology of both ML and NJ phylogenetic tree for miR167 family members was found to be mostly similar, except changes in position of some members (Fig. 3 and Supplementary Fig. S1). Both the ML and NJ tree showed that all of hundred and fifty three miR167s were categorized in two groups with high bootstrap value (Fig. 3 and Supplementary Fig. S1). The group I clade of ML tree supported thirty miR167 sequences and rest clustered in group II (Fig. 3). This tree shows that all the miR167s, which are processed from the 3′ end of the irrespective *pre-MIR167*, made one cluster as in group I (Fig. 3). Interestingly, the rate of divergence among the miR167s in group I was very random, which might be due to the varied substitution rates among members of group I sequences than group II.

The phylogenetic relationship in group II of ML tree was the result of multiple duplications and divergence of the sequences among the species (Fig. 3). The first divergence from common ancestral sequence was of acq-miR167 and cme-miR167e, whereas the subsequent evolutionary divergence has resulted in the separation of ath-miR167c and aly-miR167c-5p (two orthologous miR167 sequences). The branch length suggests that ath-miR167c and aly-miR167c-5p evolved faster with less substitution rate than acq-miR167 and cme-miR167e. The phylogeny places the ppt-miR167 separately in the group II, but along with most conserved miR167 sequences from the other species (Fig. 3). This indicates that the ppt-miR167, with faster and higher substitution rate, had undergone sequence diversification from others in group II. Similarly, ptc-miR167h-5p and tae-miR167b evolved faster with higher substitution rate than its own family members present in the same cluster (Fig. 3).

**Figure 4:**
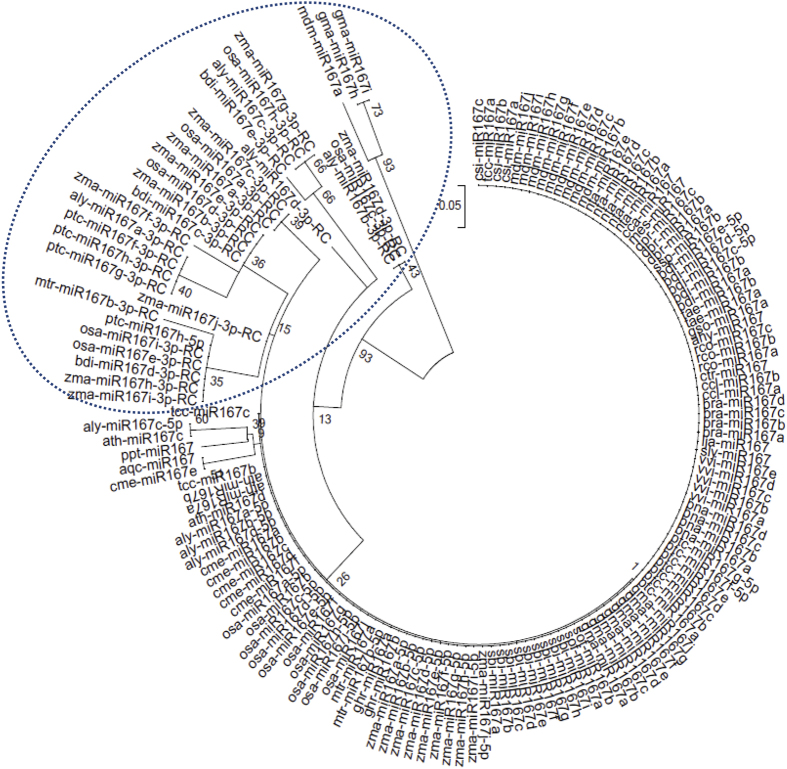
An unrooted ML phylogeny of miR167s along with reverse complementary sequences of miR167-3p using MEGA5. This tree is similar to Fig. 3, but contains reverse complementary sequences of miR167-3p (highlighted, encircled portion) along with three miR167-5ps namely, gma-miR167h/i and mdm-miR167a. All other miRNA167s (−5p) belong to group II. The scale bar represents the nucleotide substitution rate.

**Figure 5:**
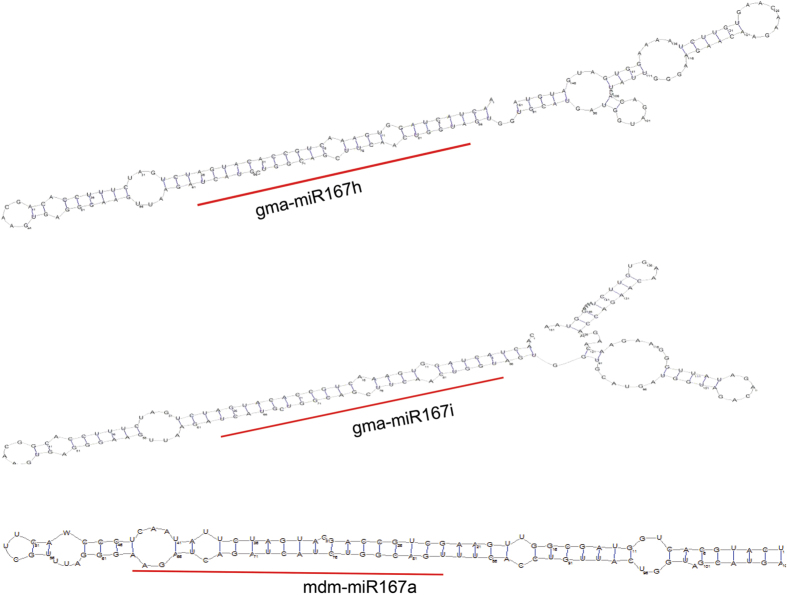
Stem-loop structures of three *MIR167* precursor sequences. (**A**) Secondary structure of gma-miR167h. (**B**) Secondary structure of gma-miR167i. (**C**) Secondary structure of mdm-miR167i. In all secondary structures, miRNAs are produced from 3′ arm of the stem-loop sequence. The miRNAs are marked by black bars. The secondary structure was predicted by using RNAshapes.

In group I, gma-miR167h and gma-miR167i have been separated from all other miR167 members in that species (Group II), and made a single cluster in group I (Fig. 3). Similarly, mdm-miR167a clustered with ptc-miR167f-3p, ptc-miR167g-3p and ptc-miR167h-3p (Fig. 3). The gma-miR167h and gma-miR167i map to Gm10: 46574263-46574413 [+], Gm20: 37901842-37901992 [−] chromosomal scaffolds respectively, whereas there is no genomic context annotated to mdm-miR167a (http://www.phytozome.org/). The two separate scaffolds revealed that the gma-miR167h and gma-miR167i are neither polycistronic nor formed from alternative splicing to share the same nucleotide sequences. The clustering of these sequences in group I, where all the miR167 sequences are processed from 3′ end of stem loop precursors, suggests that these sequences are also processed from 3′ end. The miR167-3ps are thought to be processed from 3′ end of stem loop precursors, which are complementary to miR167-5p counterparts (Fig. 4). Therefore, a separate phylogenetic tree was reconstructed using reverse complementary sequences of miR167-3ps (miR167-3p-RC) along with other miR167s (Fig. 4). The results show that miR167-3p-RC sequences have also clustered together (Fig. 4) as their corresponding miR167-3p sequences (Fig. 3). Interestingly, only ptc-miR167h-5p clustered with osa-miR167e/i-3p-RC, zma-miR167h/i-3p-RC and bdi-miR167d-3p-RC (Fig. 4). Sequences in group-I (Fig. 3) show that gma-miR167h/i and mdm-miR167a are distantly separated, but in Fig. 4, these three are clustered in group I and separated from group II consisting of all other miR167 family members of the respective species. We have further cross verified the precursor *MIR167* sequences by Mfold^44^ and RNAshape software tools and found that gma-miR167h, gma-miR167i and mdm-miR167a are processed from the 3′ end of their respective precursor sequences (Fig. 5).

The ClustalW alignment in the MEGA5 shows many unique miR167 sequences; each unique mature miR167 sequence may be derived from multiple precursor sequences (Fig. 1). We have used the *psRNATarget* web tool^46^ for the identification of the novel target of the unique miR167 (Table 2). We have predicted that the unique miR167 sequences might target genes other than *ARF6* and *ARF8*, which are the proved targets of miR167 in *A. thaliana*. Our analysis suggests that gma-miR167h targets *METHYL-CROTONOYL-CoA CARBOXYLASE* (*MCCC*), which is not homologous to any *ARF*s, as observed through BLAST, indicating the possible functional diversification of gma-miR167s in *G. max* (Table 2). Similarly, we found that *LEUCINE-RICH REPEAT RECEPTOR-LIKE PROTEIN KINASE* (*LRPK*) gene is the predicted target of gma-miR167i, which has diverged most among analyzed miR167s. We have also found *CALCINEURIN B-LIKE10* (*CNBL*) to be a new target of mdm-miR167a, which has diverged from all other mdm-miR167s (Table 2, Fig. 3). Our study suggests that these variations in targets are due to the sequence diversification of miR167s processed from 3′ end of the precursors. However, we cannot rule out the existence of additional novel targets, which might be uncovered once more refined genome sequences are available.

### Phylogenetic analysis of *pre*-*MIR167* sequences

**Figure 6:**
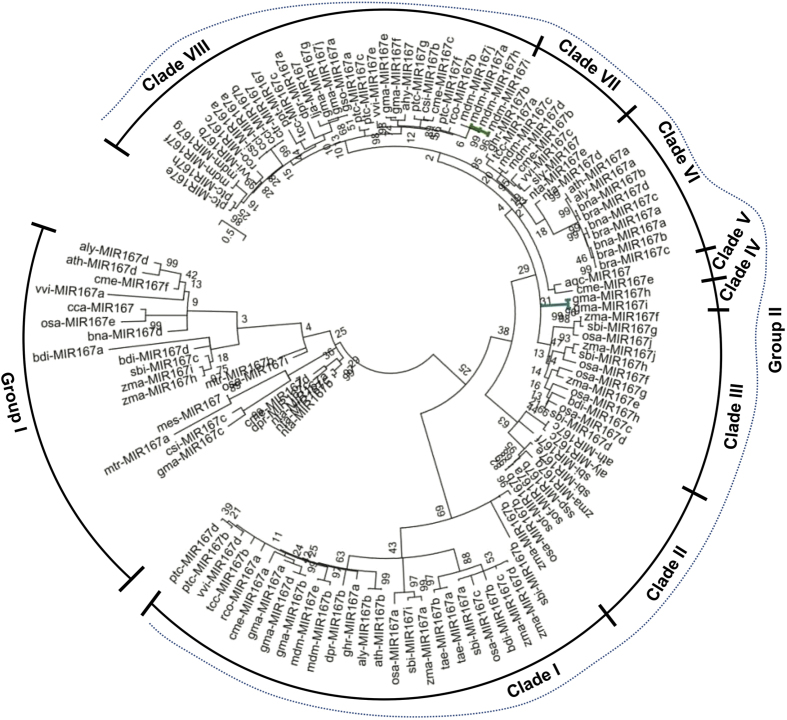
An unrooted ML phylogeny of *MIR167s* using MEGA5. The tree is divided into two groups as group I and group II (marked with dotted lines). The group I supports twenty three *MIR167s* whereas group II is classified into I, II, III, IV, V, VI, VII and VIII clades containing rest of the *MIR167s*.

The *pre-MIR167s* showed variations in divergence and clustering of sequences. This is due to the less conservation of *pre-MIR167* of sequences, as shown in the alignment statistics (Fig. 2). Though precursor sequences are less conserved than the mature miR167s, our analysis was aimed to find the evolutionary pattern among the different *pre-MIR167s* from different species. The ML tree is divided into two groups, Group I (comprising of twenty three *pre-MIR167* sequences) and remaining sequences in Group II (Fig. 6). Unlike its mature miR167s, which are mostly conserved, the *pre-MIR167* sequences of *A. thaliana (ath-pre-MIR167)* and *A. lyrata (aly-pre-MIR167)* are distributed in four clusters in both group I and II, and have diverged more (Fig. 6). In the group I, *ath-pre-MIR167d* and *aly-pre-MIR167d* made one cluster. Among all four *pre-MIR167s* from both *A. thaliana* and *A. lyrata*, only *ath-pre-MIR167b* and *aly-pre-MIR167b* clustered in clade I. The sequences in group I has diverged in three branches, one branch consisting of five *pre-MIR167s* namely *nta-pre-MIR167a, nta-pre-MIR167b, nta-pre-MIR167c, dpr-pre-MIR167c* and *cme-pre-MIR167d*, whereas three sequences of *Nicotiana tabacum* – *nta*-*pre-MIR167a/b/c* made a single cluster (Fig. 6). The precursor sequences of *nta-pre-MIR167b* and *nta-pre-MIR167c* are placed as the paralogous to the *nta-pre-MIR167a*. The GeneDoc analysis showed that the *nta-pre-MIR167b* has 41% sequence identity with the *nta-pre-MIR167c*, whereas *nta-pre-MIR167a* has 39% sequence similarity with these two sequences respectively (Fig. 2). Similar paralogous sequences like *zma-pre-MIR167h* and *zma-pre-MIR167i* are also found in group I. Among ten rice *osa-pre-MIR167* sequences, only *osa-pre-MIR167e* is found in group I with higher substitution rate (Fig. 6). Likewise, *vvi-pre-MIR167a, bdi-pre-MIR167a* and *mtr-pre-MIR167a* having longer branch length imply their faster evolution with higher rate of substitution in group I. The divergence of *pre-MIR167s,* within the group I, is very discrete among species (Fig. 6).

The group II of the phylogenetic tree has branched off multiple times to produce many clades (Fig. 6). The group II *pre-MIR167s* are divided into seven sub groups or clades, each group comprising highly variable number of *pre-MIR167s* (in parentheses) – I (28), II (8), III (12), IV (2), V (2), VI (10), VII (8), VIII (32). The clade I of group II has further diverged through the duplication of sequences in the evolutionary process to a major sub-clade consisting of twenty five *pre-MIR167* genes. Interestingly, maximum number of *pre-MIR167s* in clade I are *pre-MIR167b* and *pre-MIR167a* from multiple species. There are five conserved paralogous pairs of *pre-MIR167* like *zma-pre-MIR167c/d, tae-pre-MIR167a/b, mdm-pre-MIR167b/e, ath-pre-MIR167b* and *aly-pre-MIR167b, gma-pre-MIR167b/d* (sister to *gma-pre-MIR167a*) and *ptc-pre-MIR167b/d* (Fig. 6, clade I). Three conserved precursors namely *sbi-pre-MIR167b, zma-pre-MIR167b*, and *osa-pre-MIR167b* formed a separate cluster. In clade II, the precursor sequence *zma-pre-MIR167g* has diverged with higher rate of substitution from *ssp-pre-MIR167b, sof-pre-MIR167a* (which also have conserved mature miR167 sequences; Fig. 3) and *sof-pre-MIR167b*, which are separated from *sbi-pre-MIR167e* (Fig. 6). On the other hand, *sbi-pre-MIR167f* and *ath-/aly-pre-MIR167c* have diverged separately from a common ancestor. Likewise, in clade III, *sbi-pre-MIR167d, osa-pre-MIR167d, bdi-pre-MIR167c, osa-pre-Mir167h, zma-pre-MIR167e, osa-pre-MIR167g, osa-pre-MIR167f, sbi-pre-MIR167h*, and *zma-pre-MIR167j* have a common ancestor, sharing with *zma-pre-MIR167f, sbi-pre-MIR167g* and *osa-pre-MIR167j*. Interestingly, we have observed that the *gma-pre-MIR167h* and *gma-pre-MIR167i* are conserved and fall in clade IV within the group II, which is similar to their mature gma-miR167h and gma-miR167i. Likewise, *aqc-pre-MIR167c* and *cme-pre-MIR167e* (clade V) have formed a separate cluster (Fig. 6). The clade VI of group II, which consists of *pre-MIR167s,* has made four clusters. We have also observed that two conserved *pre-MIR167*s, namely *ath-pre-MIR167a* and *aly-pre-MIR167a,* shares the common ancestor with the other two plant *pre-MIR167* precursors of *Brassica rapa* (*bra-pre-MIR167s*) and *Brassica napus* (*bna-pre-MIR167s*). Interestingly, all the precursors of these two plant species have clustered together, and these orthologous *pre-MIR167s* have shared a common ancestor. Within clade VII, *mdm-pre-MIR167c* and *d* are produced from a single cluster, which has three sister branches comprised of five other *pre-MIR167s* from different species. One such branch has *ghr-pre-MIR167b* diverged from *tcc-pre-MIR167a* with faster rate of substitution. Likewise, another branch has *cme-pre-MIR167b* diverged from *vvi-pre-MIR167c* and *sly-pre-MIR167*. These three clusters were sharing a common ancestor with *nta-MIR167e* (Fig. 6). The *MIR167* precursors of clade VIII have diverged into three branches, where the two major branches are *mdm-pre-MIR167a/h/i* and *mdm-pre-MIR167g/f*. Here, *mdm-pre-MIR167a/h/i* has diverged from *mdm-pre-MIR167j* and *mdm-pre-MIR167g/f* has diverged from *ptc-pre-MIR167e/h* with higher rate of substitution. In the sub-clade of *mdm-pre-MIR167a/h/i, ahy-pre-MIR167* has evolved with higher rate of substitution from *gma-pre-MIR167e/f*. In another sub-clade, *mdm-pre-MIR167g/f, gma-pre-MIR167g/h* and *gso-pre-MIR167a* have evolved from a common ancestor. The *csi-pre-MIR167a, ctr-pre-MIR167* and *ccl-pre-MIR167a/b* also have a common ancestor. The *gma-pre-MIR167g/h* and *gso-pre-MIR167a* have diverged from *lja-pre-MIR167*. One of the major branches among them has no sub-clade containing only *ptc-pre-MIR167a/c*, whereas former one evolved faster with higher rate of substitution (Fig. 6).

### Identification of Unique miR167 and Unique Target genes

Using multiple sequence alignment (done with ClustalX2) of mature miR167s of all species (selected for this study), we have grouped miRNAs according to their sequence similarity and uniqueness. A total of 14 unique miR167 (UmiR167-1 to UmiR167-14) sequences have been identified, where 6 UmiR167s are shared by multiple species, 2 UmiR167 have more than 1 miR167 sequences of same species, and 6 UmiR167 sequences have only one miR167 sequence (Table 2). The largest number of miR167 sequences (54) from 21 different species has the UmiR167-1 sequence, which has 3 UTSs (Unique Target Sequences), and is predicted to target transcripts from total 19 genes (Table 2). Likewise, UmiR167-2 sequence, shared by 36 miR167s from 15 species, has 3 UTSs possessed by only 3 target genes (Table 2). Though 8 miR167 sequences from 6 species share UmiR167-3, it has only one UTS belonging to one target gene. Similarly, UmiR167-6, shared by 6 miR167 sequences from 4 species, is having only 1 UTS, and it targets 6 genes (Table 2). Interestingly, only 2 miR167 sequences share the UmiR167-4 from 2 species and is having 5 UTSs belonging to 6 target genes. The UmiR167-5 is shared by 7 miR167 sequences from 5 species and has 6 UTSs belonging to 7 target genes. The UmiR167-10 is shared by bna-miR167a/b and UmiR167-13 is shared by mdm-miR167h/i/j within their respective species *B. napa* and *M. domestica* only. Furthermore, each of UmiR167-10 and UmiR167-13 has only 1 UTS, but they target 4 and 2 genes, respectively (Table 2). Each of other UmiR167s (UmiR167-7/8/9/11/12/14) are shared by only 1 miR167 sequence and also targets 1 gene except the UmiR167-12 (targeting 2 genes).

A unique miR167 sequence may target transcripts of many genes, which may share the same miR167 complementary site (unique target sequence) despite their sequence variation at the whole gene level. On the other hand, conserved target genes may undergo sequence variation in the miR167 binding sites. Therefore, the uniqueness and sequence variation of miR167 sequences (on the basis of similarity) as well as their complementary target sequences are very important to understand the coevolutionary pattern of miR167s, their corresponding targets, and miR167 mediated gene regulation.

We have identified a total of 14 UmiR167s using ClustalW from MEGA5. Further, each of 14 UmiR167 sequences have been used for the identification of their complementary target sequences for its binding site using *psRNATarget* tool. Based on ‘E’ and ‘UPE’ (maximum energy to unpair the target site) value from *psRNATarget* tool, 27 UTSs have been identified for these UmiR167s (Table 2). Although 27 UTSs have been identified for 14 UmiR167s, *psRNATarget* tool predicted only 20 UTSs belonging to 11 genes targeted by 7 UmiR167s (UmiR167-1/2/4/5/7/9/12). Rest of the 7 UTSs, complementary to 7 UmiR167s (UmiR167-3/6/8/10/11/13/14), do not belong to any predicted target genes as found by *psRNATarget* tool. Therefore, by using NCBI BLAST (http://blast.ncbi.nlm.nih.gov/Blast.cgi) analysis with 100% query coverage and maximum identity, we have identified 11 putative target genes having above mentioned 7 UTSs targeted by 7 UmiR167s. Additionally, 22 orthologous sequences (putative additional targets) of target genes have also been identified using the NCBI BLAST with aforesaid parameter (Table 2).

Since in *Oryza sativa* (japonica cultivar-group), we have found two *ARF*s (*Osa-ARF6* and *Osa-ARF*) targeted by UmiR167-1 (Table 2), we have also searched for their homologs in *Osa* (indica cultivar-group) using available sequence data. Surprisingly, we have not found any homologs of these japonica *Osa-ARF6* and *Osa-ARF* in indica cultivars. Therefore, we have also searched genes targeted by UmiR167-1 in the database of *Osa* (indica cultivar-group) using nucleotide blast (http://blast.ncbi.nlm.nih.gov/Blast.cgi). Again, we couldn’t get any target gene but their similarity with the precursor of miR167s (indica). Although in japonica cultivar-group, *ARFs* were present and targeted by UmiR167-1, the *Osa-NBS-LRR, Osa-DUF630* and *Osa-RDH14* have been found to be targeted by UmiR167-2. Interestingly, these *Osa-NBS-LRR* and *Osa-RDH14* have shown resemblance with genes having accession no. EF642483.1 and LOC_Os03g02460 (from indica cultivars) respectively, and these were also potentially targeted by UmiR167-2 (Supplementary Figure S2). In case of *Zea mays (Zma)*, the nucleotide Blast searched for target genes against UmiR167s in all sequenced cultivars present in NCBI database.

### Phylogenetic analysis of Target sequences and genes

**Figure 7:**
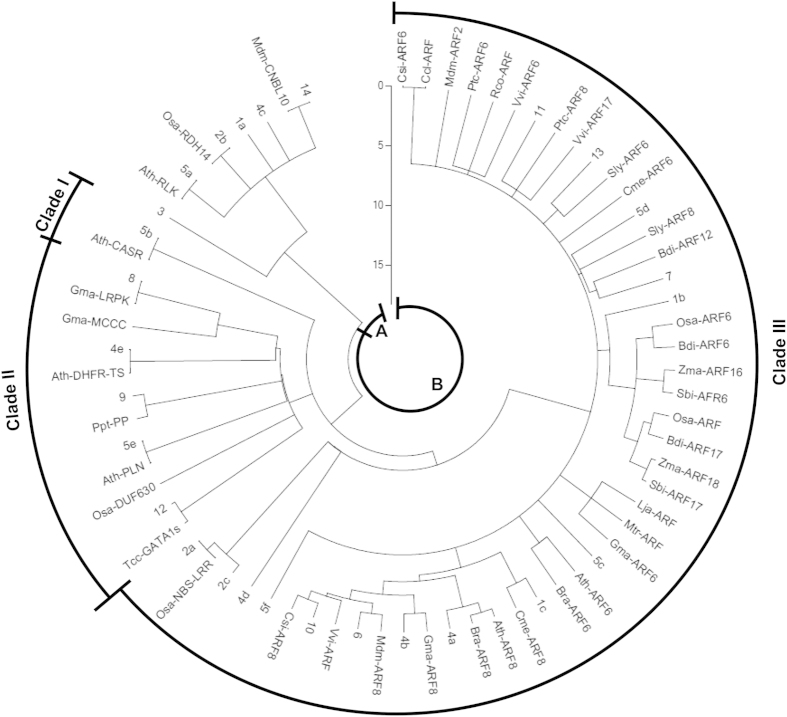
ML Phylogeny of corresponding target sequences and miR167 using MEGA5 to show relatedness. The tree is divided into two groups (**A**,**B**). Group (**B**) is divided into clade I, II and III. The numbers represent unique target sequences (UTSs) present in species specific *ARFs* and other *non-ARF* target sequences. The numbers of UTSs correspond to unique miR167s (Table 2).

To understand conservation and diversification of miR167 target sites and the coevolution of miR167s and their target genes, we have done phylogenetic analysis with 27 UTSs along with total 44 selected target genes of 14 UmiR167s. The resultant phylogenetic tree is divided into 2 groups (group A and B); group B is divided into 3 clades namely clade I, II and III (Fig. 7). The tree shows that clade III has maximum number of orthologous target genes divided into sub-clades.

In the phylogenetic tree (Fig. 7), the UTS 3 in group A is placed as an outgroup and is separated from the similar UTSs - 14, 4c, 1a, 2b, and 5a. Whereas, the UTS 14, 2b and 5a is very close to target gene *Mdm-CNBL10, Osa-RETINOL DEHYDROGENASE14* (*Osa-RDH14*) and *Ath-RECEPTOR-LIKE PROTEIN KINASE* (*Ath-RLK*), respectively (Fig. 7; Table 2). In group B, the clade I is the smallest cluster consisting of only UTS 5b, which is close to target gene *Ath*-*CALCIUM SENSING RECEPTOR* (*Ath-CASR*) (Fig. 7; Table 2). In the clade II (group B), UTS 12 is very close to target gene *Tcc*-*GATA PROTEIN ISOFORM1* (*Tcc-GATA1s*) in comparison to *Osa-DUF630*, where both are separated from a common ancestor along with another cluster which is further divided into 3 branches (Fig. 7). Out of 3 branches, one has UTS 5e showing closeness towards *Ath-P-LOOP CONTAINING NUCLEOSIDE TRIPHOSPHATE HYDROLASES* (*Ath-PLN*) superfamily gene (Fig. 7; Table 2). Another one has UTS 9, which is very close to target gene *Ppt-Predicted protein* (*Ppt-Pp*) and the last branch is separated into two clusters - one has UTS 4e close to *Ath-BIFUNCTIONAL DIHYDROFOLATE REDUCTASE/THYMIDYLATE SYNTHASE* (*Ath-DHFR-TS*) gene and UTS 8 is very close to target *Gma-LRPK* gene, which is also closely related to *Gma-MCCC* gene (Fig. 7; Table 2). Interestingly, the clade III of group B, the largest clade, has all orthologs of target genes *ARF6/8* along with *Osa-NBS-LRR DISEASE RESISTANCE PROTEIN* (*Osa-NBS-LRR)* gene (Fig. 7). First division in clade III has given 3 branches, separated from the common ancestor (Fig. 7). The first branch is further separated UTSs 2a and 2c, whereas UTS 2a has closeness towards target gene *Osa-NBS-LRR* (Fig. 7; Table 2). The second branch has only UTS 4d and the third one is divided into many branches and clusters (Fig.7). The UTSs 5f, 5c and target genes *Mdm-ARF2, Cme-ARF6* are separated and distantly placed in the clade III without forming any specific cluster. The UTS 5c is close to cluster consisting of *Ath-ARF6, Bra-ARF6* and the cluster consisting of *Gma-ARF6, Mtr-ARF*, and *Lja-ARF* (Fig. 7). The target genes *Mdm-ARF2, Cme-ARF6, Csi-ARF6, Ccl-ARF, Ptc-ARF6, Rco-ARF*, and *Vvi-ARF6* do not have any closely related UTSs but the nearest UTS 11, which is very close to target genes *Ptc-ARF8* and *Vvi-ARF17* (Fig. 7). The largest cluster in this clade has UTSs 1c, 4a, 4b, 6, 10 are closely related to target genes *Cme-ARF8, Bra- ARF8* and *Ath-ARF8, Gma-ARF8, Mdm-ARF8, Csi-ARF8* and *Vvi-ARF*, respectively (Fig. 7). Another cluster of target genes *Bdi-ARF6, Bdi-ARF17, Osa-ARF6, Osa-ARF, Sbi-ARF6, Sbi-ARF17, Zma-ARF16* and *Zma-ARF18* are much closed to only UTS 1b indicating the conserved target sites. There are some small clusters of UTSs and target gene in this clade III. The , UTS 13 is close to *Sly-ARF6*, UTS 7 is very close to *Bdi-ARF12,* and UTS 5d is close to *Sly-ARF8* as well as *Cme-ARF6* (Fig. 7).

### Validation of novel non-conserved target of miR167

**Figure 8:**
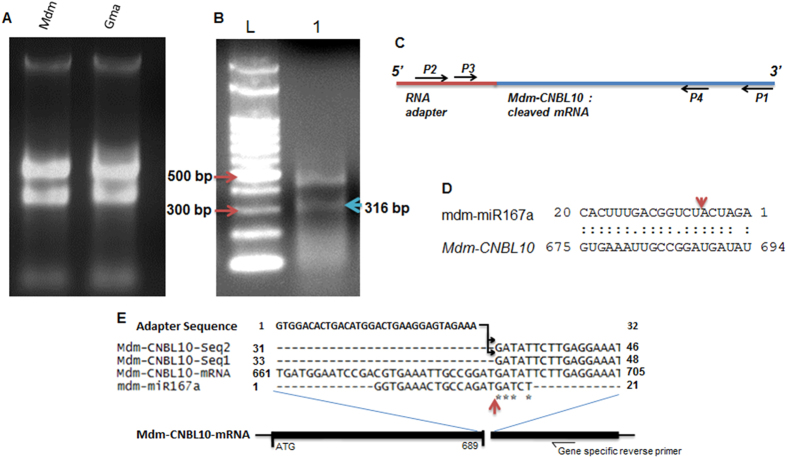
5′RLM-RACE PCR based validation of novel miR167 targets. (**A**) Isolated total RNA from seeds of *Malus domestica* (variety golden delicious, leaves from different cultivar not shown here) and *Glycine max* seedling, (**B**) RLM-RACE PCR showing cleaved product of desired size (316 bp) from *Mdm-CNBL10* (lane 1), lane L indicate DNA ladder, (**C**) Schematic presentation of RLM-RACE as in “B”: blue line indicate cleaved target mRNA, red line indicate RNA adapter ligated to cleaved mRNA, position of oligo (dT) (p1), adapter forward primer (p2), nested adapter primer (p3), gene specific reverse primer (p4) are indicated with black arrows. (**D**) Complementarity between UmiR167-14 and *Mdm-CNBL10* and red arrow shows cleavage site, (**E).** Alignment of mdm-miR167a with their target gene *Mdm-CNBL10* and sequenced 5′ RLM RACE PCR products. The 5′ RLM RACE PCR products also have ligated RNA adapter. The red arrow indicates the cleavage site and * indicates the identity among the sequences.

Computational identification provided an extensive list of potential miR167 targets for diverse plant species (Table 1). It is well known that *ARF6/8* and *IAA-Ala Resistant3* *(IAR3)* are evolutionary conserved targets of miR167 in *Arabidopsis*^47^. It was evident from our phylogenetic analysis that gma-miR167h/i and mdm-miR167a are clustered with 3′ derived miR167s rather than 5′ derived miR167s (Fig. 3) and predicted to cleave non-conserved target mRNAs. We have then selected targets *Mdm-CNBL10, Gma-MCCC* and *Gma-LRPK* to validate the potential cleavage of their mRNAs through 5′RLM-RACE PCR (see materials method for details). After ligation of RNA adapter to isolated total RNA from Mdm and Gma (Fig. 8), 5′ RLM-RACE was performed and followed by PCR amplification using adapter specific forward primer and gene specific reverse primers for *Mdm-CNBL10, Gma-MCCC* and *Gma-LRPK* respectively^48^. Interestingly, we observed a desired band size of 316 bp (Fig. 8B) for a cleaved *Mdm-CNBL10* mRNA (also having ligated RNA adapter, Fig. 8C) from RACE- PCR, as we predicted. Two replicates (from independent PCR) of desired size bands were purified from gel and sequenced (Supplementary information S3A). The sequencing results confirmed the cleavage of *Mdm-CNBL10* mRNA at 689 bp downstream of ATG (including ATG) at the complementary site of mdm-miR167a (Fig. 8D–E, Supplementary information S3A). Unusually, this cleavage took place at 5^th^ position rather than 10^th^ from the 5′ end of the mdm-miR167a, probably due to the presence of a mismatch at 9^th^ position (Fig. 8D,E). However, we could not validate cleavage of *Gma-MCCC* and *Gma-LRPK* by gma-miR167h and i, by RLM-RACE. This suggests the miR167 mediated cleavage of novel *Mdm-CNBL10* target mRNA and possible translational inhibition of novel *Gma* target mRNAs (as above).

## Discussion

The miR167s have previously been shown to have important roles in plant gametophyte development and adventitious root development in *Arabidopsis* by targeting *ARF6* and *ARF8* through cellular auxin signaling^37^. Since there are no supporting experimental evidences for the evolutionary relationship of miR167 family till now, it was imperative to study the phylogenetic evolution of miR167. We have reconstructed the phylogeny of miR167 sequences for studying their sequence conservation and diversification among diverse plant species. Our analysis on evolutionary relationship among miR167 sequences shows that the mature miR167 family members, except gma-miR167i, gma-miR167h and mdm-miR167a which together produced a different group, are conserved and clustered in a single clade (Fig. 3). The conservation of mature miRNAs is due to the high level of sequence homology among miR167s. Therefore, a little change of the mature sequences in the phylogenetic tree indicates sequence diversification and the pattern of processing. Interestingly, the miRNAs represented as miR167-3p formed another group. Further verification by reconstructing the tree with the reverse complementary sequences of these miR167-3ps along with other (5p miR167s) proved that miR167-3ps are indeed separate from 5p. As we have suspected, all these miR167-3p-RCs formed a separate group from miR167-5p similar to their corresponding miR167-3p sequences (Fig. 4 and Fig. 3). This suggests that the miR167-3p mature sequences were processed from the 3′ end of their precursor sequences and represented as separate miR167-3p, rather than miR167*. So it could be assumed that not all miR167* are degraded during the processing of precursor, rather they function as miR167-3p as evident from deep sequencing results in miRBase (http://www.mirbase.org/).

Three of the mature miR167s pairs ath-miR167c/aly-miR167c, gma-miR167h/i and acq-miR167/cme-miR167e have maintained their conservation at the precursor level in clade II, IV and V, respectively (Fig. 6). The clustering of monocot *pre-MIR167s*, namely *sbi-pre-MIR167e*/*zma-pre-MIR167g, sof-pre-MIR167a/b* and *ssp-pre-MIR167b* with dicot *ath-pre-MIR167c*/*aly-pre-Mir167c* (Fig. 6, clade II) indicates the origin of *ath-pre-MIR167c* prior to monocot-dicot divergence. The separation of precursor sequences of some conserved mature miR167 sequences in precursor phylogenetic tree such as ath-miR167a/b, sbi-miR167, zma-miR167, ptc-miR167 etc. are due to more sequence diversification in the level of precursor sequence (Fig. 3 and Fig. 6). It could also be due to the long precursor sequences (*pre-MIR167s*), which are subjected to more mutational events than much smaller (18–21 nucleotides) mature sequences (miR167s). Similarly, the diversity in sequences (within the mature miRNA part of some species such as gma-miR167h/i and mdm-miR167a) from their corresponding other mature miR167 family members are due to critical changes in the mature sequence in the course of evolution (Table 2). This is also evident from our phylogenetic analysis that gma-miR167h/i and mdm-miR167a are not clustered with 5′ derived miR167s, however, clustered with 3′ derived miR167s (Fig. 3), whilst in the phylogenetic analysis of *pre-MIR167s* these *gma-pre-MIR167h/i* and *mdm-pre-MIR167a* are clustered close to those *pre-MIR167s* which give rise to 3′ derived miR167s (Fig. 6).

Our analysis indicates that the number of sequence duplication events were high in *Glycine max* (gma), *Malus domestica* (mdm), *Oryza sativa* (osa), *Zea mays* (zma), *Sorghum bicolor* (sbi) and *Populous trichocarpa* (ptc) (Table 1). All mature gma-miR167s (a–g), except gma-miR167i/h, are conserved and clustered together in group II (Fig. 3). Although precursor *gma-pre-MIR167s* are clustered in group II, their conservation is discrete and appeared in different clades such as *gma-pre-MIR167a/b/d, gma-pre-MIR167h/i* and *gma-pre-MIR167e/f/g/j* in clades I, IV and VIII, respectively (Fig. 6). This is due to the diversification with a different rate of substitution in precursor genes during evolution. Similar pattern of evolution was observed among mature and precursor mdm-miR167s. Except mdm-miR167a, all other mdm-miR167b–j were conserved and clustered in group II. At precursor level, *mdm-pre-MIR167b/e, mdm-pre-MIR167c/d* and *mdm-pre-MIR167a/f/g/h/i/j* have clustered in clade I, VII and VIII, respectively (Fig. 6). We have observed huge variation in the length of *mdm-pre-MIR167* sequences, which is another cause of their diversification at precursor level in addition to the variation in the present sequences. Deletion or addition of sequences during duplication process might have caused the changes in the length of precursors/genes during evolution of *mdm-pre-MIR167* family. Interestingly, all osa-miR167s/−5ps, except osa-miR167a, are highly conserved, and present in group II (Fig. 3). However, precursors *osa-pre-MIR167a/b/c* have clustered discretely within clade I, *osa-pre-MIR167d/f/g/h/j* clustered in clade III of group II and *osa-pre-MIR167e/i* clustered in group I (Fig. 6). On the other hand, all zma-miR167a–j (−5p) are highly conserved and clustered in group II (Fig. 3), while their precursors are clustered in group I and II (clade I–III). Similarly, mature sbi-miR167a-i are highly conserved and clustered in group II and their precursors also have followed the similar pattern as in case of *osa-pre-MIR167s*. This finding implies that despite large number and plausibly high duplication rate among the *osa-, zma-* and *sbi-pre-MIR167s*, the divergence among the respective mature sequences was very low, even though their precursor sequences have highly diverged, in these monocot species. Further, predicted target genes of these monocot miR167s, orthologs of *ARF*s, are also found to be conserved and appeared in a single cluster (clade III of group B) and are close to the UTS 1b (Fig. 7). This cluster of conserved monocot *ARFs* are further branched into three clusters in the clade III of group B, where one cluster consists of *Zma-ARF18, Sbi-ARF17, Osa-ARF*, and *Bdi-ARF17*, the second one consists of *Zma-ARF16* and *Sbi-ARF6,* and the last one has *Osa-ARF6* and *Bdi-ARF6* . Therefore, we suggest that in monocots (except *O. sativa*), miR167 mediated gene regulation is least affected during the course of evolution resulting into their functional conservation including auxin signaling, (Fig. 7). It is likely human selection or domestication has contributed to this evolutionary pattern among these crops. Interestingly, our phylogenetic analysis suggests that sequences of *Brassica rapa* is conserved at mature as well as precursor (miR167/MIR167) level and the event of duplication had occurred with a slow rate of substitution, since their precursor sequences are of almost equal length with maximum similarity. Not only at mature and precursor level, but target genes of bra-miR167 are also conserved. The phylogenetic analysis of UTSs and target genes have shown that *Bra-ARF6* is clustered with *Ath-ARF6* which is closely related with UTS 5c and *Bra-ARF8* is clustered with *Ath-ARF8* which is closely related to UTS 4a (clade III of group B; Fig. 7). Phylogenetic similarity and absence of any new predicted non-ARF6/8 targets, suggest functional conservation of miR167s in *Brassica*.

Previous experimental studies on *Arabidopsis* miR167^37^ have reported that over expression of only ath-miR167a (among four ath-miR167s) showed arrested flower development, similar to mutants of target *arf6-2* and *arf8-3* plants. This suggests that there is a prime requirement of miR167a to be conserved in the diverse plant species. Interestingly, this has been reflected in our phylogenetic analysis of mature miRNAs (Fig. 3), where group I caries all of the miR167a but one exception - mdm-miR167a. The clustering of the *mdm-pre-MIR167a* with the other precursor sequence of apple (*mdm-MIR167*) species such as *mdm-pre-MIR167j, h, i* (Fig. 6, clade VIII) suggests that the members of the *pre-MIR167* have evolved through probable duplication of same *pre-MIR167* sequence and exist as ortholog or homolog in other species^49^. Recent study using high osmotic stress in *Arabidopsis* has shown that^47^ ath-miR167 also targets *IAR3*, an evolutionary conserved target, other than *ARF6* and *ARF8,* which suggests that there might be additional target(/s) other than targets validated in natural/control condition. This suggests that changes in the spatiotemporal expression of miRNA or predicted target genes under stress or treatment may lead to validation of additional miR167 mediated target cleavage and regulation of biological processes. Our study, using *psRNATarget*^46^ predicted that some miR167s are able to bind to different target mRNA of gene other than *ARF* (Fig. 7; Table 2)^37^. This functional diversification is caused by mutation in critical region of mature miR167 sequences, as we have earlier shown for ppt-miR166m^44^. It is evident from our analysis that *ARF6* and *ARF8,* the natural targets of miR167s, have also undergone functional diversification during the course of evolution, even though they have overlapping function^50^.

Our analysis for finding target genes of UmiR167s through *psRNATarget* tool and NCBI BLAST, predicted 27 UTSs and 44 target genes for 14 UmiR167s (Table 2). This result suggests that an UmiR167 may target one or more genes among total 44 identified candidates. In course of evolution of miR167 sequences, the complementary sequence of target genes has also been subjected to evolutionary selection pressure. Variation in either of miR167 or its complementary target sequence may lead to functional diversity of miR167 mediated regulatory processes (Fig. 7).

Using UTS and target genes, our phylogenetic analysis has predicted a total of 12 new targets and it supports the functional diversification of targets from *ARF6/8* (Fig. 7). Importantly, these new targets are not homologs of *ARF6/8,* which is evident from the phylogenetic tree, as all orthologs of *ARF6/8* are clustered separately (clustered in clade III of group B) from the newer targets except *Osa-NBS-LRR* (Fig. 7). Some of the novel targets, such as *Gma-MCCC, Gma-LRPK etc,* are closely related with UTS 8 specific to gma-miR167i. Similarly, target *Mdm-CNBL10* is closely related to UTS 14 specific to mdm-miR167a (Fig. 7). This might be due to the separate evolution pattern of gma-miR167h/i and mdm-miR167a, because these were processed from 3′ end. Therefore, they have shown different predicted target genes, leading to functional diversification.

Our phylogenomic analysis has suggested that gma-miR167h/i and mdm-miR167a are processed from 3′ end of their precursors, rather than conventionally known processing from 5′ end. This suggests that possible evolution of miRNA processing mechanism have taken place in some species like *Gma* and *Mdm,* which has contributed to functional diversification of miR167s in course of time (Fig. 7). We have predicted that these miR167s target novel genes *Gma-MCCC* and *Gma-LRPK* in soybean and *Mdm-CNBL10* in apple. Validation of mdm-miR167a mediated cleavage of *Mdm-CNBL10* through 5′RLM-RACE PCR confirms its functional diversification (Fig. 8B–E). It has been earlier reported in *Arabidopsis* that CBL10 protein, a homolog of *Mdm-CNBL10*, acts as a calcium sensor and involved in the signaling pathway during growth and development in response to salt and drought stresses. It has important regulatory role in salt tolerance as well as regulation of *ARABIDOPSIS K*^+^ *TRANSPORTER 1* (*AKT*) gene^51^,^52^. Although this gene is not regulated by miR167 in *Arabidopsis*, the *Mdm-CNBL10* homolog is targeted by mdm-miR167a due to sequence diversification in both miRNA and target site. This is likely to provide functional diversification of miR167 mediated gene regulation and stress response in apple. The predicted novel targets *Gma-MCCC* and *Gma-LRPK* could not be validated by RACE-PCR. This could be due to the fact that these targets do not express in the used cultivars (other than sequenced one; see materials and methods), or due to the translational inhibition of targets rather than cleavage^20^. Normally, full complementarity of miRNA with target mRNA ensures their cleavage. Since both gma-miR167h/i (UmiR167-7 and UmiR167-8) have only 77% and 85% complementarity with target *Gma-MCCC* and *Gma-LRPK* respectively (Fig. 8E–F), we cannot rule out the translational inhibition of these targets, instead of predicted cleavage. Despite their nature of regulation, their functional diversification remains plausible.

Other completely new targets *Osa*-*NBS-LRR* and *Osa*-*RDH14* have closeness towards UTSs 2a and 2b, respectively. However, it has also been found that the UTS 2c is very close to the cluster of UTS 2a and target *Osa*-*NBS-LRR*, showing the functional diversification of target genes, binding sites of UmiR167-2 in *O. sativa*, though *Osa-ARF* and *ARF6* are also present and have shown closeness towards the UTS 1b (Fig. 7). The absence of homologs of japonica *Osa-ARF6* and *Osa-ARF* (targets of japonica UmiR167-1) (Table 2) in indica cultivars suggest the functional diversification of miR167 regulated *ARFs*. This could be due to the change in genome sequence during human selection or domestication of rice cultivars. However, some level of functional conservation is evidenced by conservation of the UmiR167-2 and its target the *Osa-NBS-LRR, Osa-DUF630* and *Osa-RDH14* between japonica and indica rice cultivars (Supplementary Figure S2). Even in *Arabidopsis*, new target genes *Ath*-*RLK, Ath-CASR, Ath*-*PLN* and *Ath*-*DHFR-TS*, which are predicted by *psRNATarget* tool, have shown closeness toward UTSs 5a, 5b, 5e, and 4e, respectively (Fig. 7). Our phylogenetic analysis concludes that some of the UmiR167s are completely targeting new genes like UmiR167-2 (UTSs 2a–c in *O. sativa*), UmiR167-3 (UTS 3 in *T. cacao)*, UmiR167-5 (5a/b/e in *A. thaliana*), UmiR167-7 and UmiR167-8 (UTS 7 and 8 in *G. max*), UmiR167-9 (UTS 9 in *P. patens*) and UmiR167-14 (UTS 14 in *M. domestica*). This suggests that besides miR167 sequences, the common target *ARF*s have also undergone sequence diversification resulting in *ARF6/8* that are not targeted by miR167s in some cases. We have also observed an interesting result from our analysis about the functional diversification of target gene from *ARF6/8* in *T. cacao*. The UmiR167-3 targets *Tcc-GATA1s* and it is reported in *A. thaliana* that two paralogous genes of GATA family transcription factor *GATA NITRATE-INDUCIBLE CARBON-METABOLISM INVOLVED* (*GNC*) and *GNC-LIKE* (*GNL*), which are important transcriptional targets of the GA signaling pathway, are also critically regulated downstream of auxin signaling^53^. Our results further support the previous study that duplicated genes are more prone as a target of miRNAs in comparison to singletons in *A. thaliana* and these duplicated genes have shown more divergence^54^. Further, it is evident from the phylogenetic tree of UTSs and target genes that some of the UmiR167s (UmiR167-1, UmiR167-2 etc.) have more than one UTSs and target genes which belong to different clusters (Fig. 7). This suggests that instead of sequence diversification, more than one UTSs are targeted and regulated by the same UmiR167. Therefore, coevolution of both miR167 and their respective target sequences played important role in the functional diversification among diverse species.

## Materials and Methods

### Identification of miR167s and their precursor sequences

To identify the number of miR167 sequences available, we used the miRNA registry database (miRBase version 19, http://microrna.sanger.ac.uk/). The key word “miR167” was used as query against miRBase to search the miR167 family members in each plant species. We retrieved one hundred and fifty three mature miR167 and their precursor *MIR167* (*pre-MIR167*) sequences from thirty three diverse plant species including basal plants like moss (*Physcomitrella patens*), monocot (*Oryza sativa, Zea mays* etc.) and eudicot plants (*Arabidopsis thaliana, Brassica rapa* etc.) (Table 1). The nomenclature for species used for this study is in accordance with miRBase such as for *Arabidopsis thaliana* as “ath” (Table 1). The miR167 entries in miRBase were further verified using BLAST search in NCBI, (http://www.ncbi.nlm.nih.gov/), Phytozome (http://www.phytozome.net/) and TIGR (http://rice.plantbiology.msu.edu/) and plant GDB (http://www.plantgdb.org/) database. Homologs of the query sequences in these public databases were not considered, only miRNA sequences which were registered and annotated in the miRBase registry were taken. When verifying the precursor stem-loop structures, we have followed the criteria for annotation of plant miRNAs as explained in our previous paper^45^. Since in most cases, it did not specify the arm region on the stem-loop from which miRNA were produced, we have considered that the miR167s are processed from the 3′ part of the stem loop sequences, provided they follow the canonical structure rule of miRNAs. However, sometime we found that the mature sequences were mapped to arm-loop junction of the stem loop precursor. In those cases, we have verified miR167 using both RNAshape^55^ and Mfold RNA secondary structure prediction^45^ tool, using the few parameter settings for secondary structure prediction. We have chosen those secondary structures which have at least 18 bp matching in the folded region and a central loop free energy not greater than −18 kcal/mol. Each mature miR167 sequence was carefully cross checked for its identification using the plant miRNA database web server tools and the sequence data of miR167 obtained were used in this study.

### Sequence alignment and the phylogenetic analysis

The Multiple Sequence Alignment of miR167 sequence was performed using ClustalW (Version 2.0)^56^ with default parameter settings in MEGA5 phylogenetic analysis tool^57^ (Fig. 1). Percentage Identity of aligned sequences was studied using Kalmogorov-Smirnov statistical test in GeneDoc (version 2.7) (Fig. 2)^58^. We considered all of the sites in both the mature and the precursor sequences for the maximum likelihood (ML) and neighbor joining (NJ) methods and subsequent phylogenetic tree generation for studying the comparative evolutionary process, with emphasis on ML tree. For the maximum likelihood method, GTR Substitution model was used, with a discrete Gamma distribution among sites (4 categories), and gamma distributed with Invariant sites (G + I) as rate among sites. The bootstrap consensus phylogenetic tree was inferred from 1000 replicates^59^. For the inference of tree, we used BioNJ as the initial tree for the maximum likelihood (ML) using heuristic method. Similarly, for the NJ method, maximum composite likelihood model was used along with gamma distribution site (G) as rate among sites.

The sequences of target genes, identified by either *psRNATarget* tool or by BLAST, were used for multiple sequence alignment by ClustalX2. Further, these target genes were used for phylogenetic analysis with MEGA5. The analysis was based on their distance estimation (distance matrix) method, since they didn’t show any consensus during multiple sequence alignment by ClustalX2. Other parameters used for phylogenetic analysis included the substitution model, p-distance with uniform rates, and pairwise deletion for the gap treatment.

### Target prediction

The sequences of mature miR167s of all species were aligned with the help of ClustalW (2.0) in the MEGA5 and separated according to their sequence similarity and uniqueness. These unique miR167 (UmiR167) sequences were used for the prediction of targets of miR167s from all species (analyzed here) using *psRNATarget* tool^46^. Analysis was conducted by setting maximum E-value score as four. The complementary sequences of UmiR167 with their target genes, unique miR167 binding sites (on target mRNAs) were identified and numbered (Table 2). These identified unique miR167 binding sites, known as UTS, were selected on the basis of highest UPE vale (Table 2). Although UTS and their corresponding target genes were predicted by *psRNATarget* tool, some of UmiR167s were not having any predicted UTS. Therefore, for these UmiR167s, putative target genes were searched using nucleotide blast (http://blast.ncbi.nlm.nih.gov/Blast.cgi) keeping 100% query coverage and identity, in all available plant genome taken here. The nomenclature for the target genes used in this study was specified here as “*Ath-ARF6*” to indicate *A. thaliana ARF6;* similarly it was done for *Ath-ARF8.* Same style was used for target genes from other species (as in Table 1). Further, a phylogenetic analysis was done to understand the evolutionary pattern and reveal critical sequence variation in miR167 target sites. Based on complementarity of unique miR167 sequences with their target genes, unique miR167 binding sites (on target mRNAs) were identified and numbered (as 1, 2, 3 etc.; Table 2). Phylogenetic tree was reconstructed using predicted target sequences and their unique miR167 binding sites to reveal the critical sequence variation in miRNA target sites.

### RNA isolation and target validation through 5′ RLM-RACE-PCR

A modified procedure for RNA ligase-mediated rapid amplification of cDNA ends (5′ RACE) was followed with the GeneRacer Kit (ThermoFisher Scientific/Invitrogen, CA, USA) as described previously^10^. Total RNA was isolated using modified TRIzol^®^Reagent (ThermoFischer Scientific/Ambion, USA) from the apple twig (*Malus domestica*, cultivar Vance) and *Glycine max* (hybrid cultivar JS-335xUPSM-534) shoot and root tissues^60^. Plant material from the sequenced cultivars of *Md*m (Golden delicious) and *Gma,* which was used for sequence analysis, could not be availed. Due to this, we have prepared seed specific RNA from apple fruit (Golden delicious).

To isolate apple seed specific RNA, we used a modification of the method as reported earlier^48^. Briefly, approximately 50 mg of seed tissues were homogenized in liquid nitrogen and 500 μl of extraction buffer (100 mM Tris-HCl of pH 9.0, 20 mM EDTA of pH 8.0, 150 mM NaCl, 2% SLS, 5 mM DTT) was added to it. Then equal volume of Phenol:Chloroform:Isomalyalcohol (25:24:1) was added, mixed and centrifuged at 4 °C. 350 μl of P:C:I (25:24:1) and 650 μl of Guanidine HCl buffer (8 M Guanidine HCl, 20 mM MES, 20 mM EDTA of pH 8.0, 50 mM β-mercaptoethanol) was added to the supernatant and centrifuged as above. The supernatant was similarly purified with 500 μl of chloroform. RNA was precipitated from the aqueous phase with 1/10^th^ volume of 3M sodium acetate of pH 6.0 and 2 volume of chilled 100% ethanol. After incubation at −80 °C for 1 hour, it was centrifuged at cold to precipitate, washed with 70% ethanol. Dried RNA pellet was dissolved in RNAse free water and quantified with Nanodrop 1000 (Thermo SCIENTIFIC, USA), and gel electrophoresed (Fig. 8A).

Total RNA was treated with DNase I (Fermentas, USA) as per company’s manual, purified and ligated to the GeneRacer RNA oligo adapter without any further modification. The RLM-RACE PCR was done using GeneRacer^®^Core Kit (ThermoFischer Scientific/Invitrogen, USA). The GeneRacer Oligo (dT) primer was used to prime cDNA synthesis with reverse transcriptase. Primary amplification was performed on this cDNA using GeneRacer 5′ Primer (5′-CGACTGGAGCACGAGGACACTGA-3′) and the GeneRacer 3′ Primer (5′-GCTGTCAACGATACGCTACGTAACG-3′) to generate a pool of non-gene-specific 5′ RACE products. The conditions used for this amplification step were the same as those for gene-specific RACE recommended by the manufacturer, with the exception that an extension time of 1 min 20 sec was used. Gene-specific 5′ RACE reactions were done with the GeneRacer 5′ Nested Primer and gene-specific reverse primers as follows: *Mdm-CNBL10* (LOC103440590)-(5′-CGCAATACGCAGGAGCTTTGG-3′), *Gma-MCCC* (02G145300)-(5′-CAGGTGAAGACCCGTTGATGG-3′) and *Gma-LRPK* (LOC100779669)-(5′-GCTGGATATTCGACACCGGTTG-3′). The 5′ RACE-PCR products of 316 bp size from two replicates were purified from gel with the help of FavorPrep™ GEL/PCR Purification Mini Kit (FAVORGEN, Taiwan) and sequenced (using ABI3730xl DNA analyzer available at our institute central instrument facility).

## Additional Information

**How to cite this article**: Barik, S. *et al.* Coevolution Pattern and Functional Conservation or Divergence of miR167s and their targets across Diverse Plant Species. *Sci. Rep.* **5**, 14611; doi: 10.1038/srep14611 (2015).

## Supplementary Material

Supplementary Information

## Figures and Tables

**Table 1:** List of miR167s retrieved from miRBase (version 19).

Sl. No.	miR167 IDs	Name of plant species	No. of 5′miR167s	No. on 3′end	No. detectedexperimentally
1	ath-miR167	*Arabidopsis thaliana*	4		4
2	aly-miR167	*Arabidopsis lyrata*	4	4	4
3	cme-miR167	*Cucumis melo*	6	0	0
4	osa-miR167	*Oryza sativa*	10	6	10
5	mtr-miR167	*Medicago truncatula*	2	1	2
6	ghr-miR167	*Gossypium hirsutum*	2	0	2
7	zma-miR167	*Zea mays*	10	10	10
8	sbi-miR167	*Sorghum bicolor*	9	0	9 (by homolog)
9	sof-miR167	*Saccharum officinarum*	2		2 (by homolog)
10	gma-miR167	*Glycine max*	8	2	10
11	ptc-miR167	*Populus trichocarpa*	8	3	3 (experimental)
12	ppt-miR167	*Physcomitrella patens*	1	0	0
13	bna-miR167	*Brassica napus*	4	0	4
14	vvi-miR167	*Vitis vinifera*	5	0	5
15	sly-miR167	*Solanum lycopersicum*	1	0	1
16	lja-MIR167	*Lotus japonicus*	1	0	1 (by homolog)
17	bra-miR167	*Brassica rapa*	4	0	4
18	aqc-MIR167	*Aquilegia caerulea*	1	0	0
19	ccl-miR167	*Citrus clementine*	2	0	1
20	rco-miR167	*Ricinus communis*	3	0	3
21	gso-miR167	*Glycine soja*	1	0	1
22	tae-miR167	*Triticum aestivum*	2	0	1
23	bdi-miR167	*Brachypodium distachyon*	5	3	1
24	ssp-miR167	*Saccharum* ssp.	1	0	0
25	dpr-miR167	*Digitalis purpurea*	3	0	3
26	mes-miR167	*Manihot esculenta*	1	0	0
27	cca-miR167	*Cynara cardunculus*	1	0	1
28	nta-miR167	*Nicotiana tabacum*	5	0	5
29	mdm-miR167	*Malus domestica*	10	0	10
30	csi-miR167	*Citrus sinensis*	3	0	3
31	tcc-miR167	*Theobroma cacao*	3	0	0
32	ctr-miR167	*Citrus trifoliata*	1	0	0
33	ahy-miR167	*Arachis hypogaea*	1	0	1

**Table 2:** Predicted targets of unique miR167 sequences using *psRNATarget* web server tool.

UmiR167(1–14)	miRNA ID	Unique miR167Sequence	UR	Unique TargetSequence(Total 27 UTS)	E value	TargetAccessibility(UPE)	TargetAccession	TargetDescription	Inhibition
1	ath-miR167a,b, aly-miR167a,bcme-miR167a,b,e, osa-miR167a–c mtr-miR167a,b,zma-miR167a–d sbi-miR167a,b,i,gma-miR167a,b,d ptc-miR167a–d, bna-miR167c vvi-miR167b,d,e,sly-miR167 bra-miR167a–d aqc-miR167 rco-miR167a,btae-miR167a,b bdi-miR167a,b dpr-miR167a,b nta-miR167d,emdm-miR167b–g tcc-miR167a,b.	UGAAGCUGCCAGCAUGAUCUA	**1a**	UGAUCAUUCUGGCAGCUUUG	3	19.68	AT5G41300.1	Ath-Receptor-likeprotein kinase-relatedfamily protein	Cleavage
			**1b**	AGAUCAGGCUGGCAGCUUGU	3.5	18.686	AT1G30330.1	Ath-ARF6	Cleavage
							NM_001247734.1	Sly-ARF6	
							AK339568.1	Lja-ARF	
							LOC100796447	Gma-ARF6	
							Os06g46410.1	Osa-ARF	
							Os02g06910.1	Osa-ARF6	
							HM004531.1/HM004533.1	Zma-ARF16/18	
							POPTR_0001s36900g/POPTR_0002s05590g	Ptc-ARF6/8	
							Sb04g004430.1/Sb10g027220.1	Sbi-ARF6/17	
							LOC102620318	Csi-ARF6	
							CICLE_v10014198 mg	Ccl-ARF	
							LOC103484339	Cme-ARF6	
			**1c**	UAGAUCAGGCUGGCAGCUUGU	3.5	17.28	AT5G37020.2	Ath-ARF8	Cleavage
							MTR_2g018690	Mtr-ARF	
							XM_002532937.1	Rco-ARF	
2	osa-miR167d–j, cme-miR167d,f,mtr-miR167b, zma-miR167e–j,sbi-miR167c–h, sof-miR167a,b, gma-miR167c,j,ptc-miR167e,h, vvi-miR167a, lja-miR167,gso-miR167a, ssp-miR167b, dpr-miR167c,mes-miR167, csi-miR167a,c.	UGAAGCUGCCAGCAUGAUCUG	**2a**	UAGAUCAUGCUGACAGCCUCA	2.5	13.88	Os07g29820.1	Osa- NBS-LRRdisease resistanceprotein, putative,expressed	Translation
			**2b**	UGUUCAUGCCGGCAGCUUCA	3	22.25	Os06g03830.1	Osa-retinoldehydrogenase 14,putative, expressed	Translation
			**2c**	UGAUCAUGCUGCCAGGUUCA	3.5	11.33	Os09g37520.1	Osa-DUF630	Translation
3	tcc-miR167c csi-miR167bahy-miR167, ptc-miR167f,ggma-miR167e,f , cme-miR167c	UGAAGCUGCCAGCAUGAUCUU	**3**	GAAAACAUGCUGGCAGCUUUG		N/A	TCM_042613	Tcc-GATAprotein isoform 1	N/A
4	ath-miR167c, aly-miR167c	UAAGCUGCCAGCAUGAUCUUG	**4a**	UUAGAUCAGGCUGGCAGCUUG	3.5	17.1	AT5G37020.2	Ath-ARF8	Cleavage
			**4b**	UUAGAUCAGGCUGGCAGCUUG	3.5	19.54	AT5G37020.1	Ath-ARF8	Cleavage
			**4c**	AAUGAUCAUUCUGGCAGCUUU	4	19.72	AT5G41300.1	Ath-Receptor-likeprotein kinase-relatedfamily protein	Cleavage
			**4d**	UGAGAUCAGGCUGGCAGCUUG	2.5	21.65	AT1G30330.1	Ath-ARF6	
							LOC103840407	Bra-ARF6	
			**4e**	AGGAUCAUGCUUGCCGCUUG	3.5	20.64	AT2G21550.1	Ath-Bifunctionaldihydrofolate reductase/thymidylate synthase	Translation
5	ath-miR167d, aly-miR167d,cca-miR167, nta-miR167a–c,rco-miR167c	UGAAGCUGCCAGCAUGAUCUGG	**5a**	UGAUCAUUCUGGCAGCUUUG	3	19.68	AT5G41300.1	Ath-Receptor-likeprotein kinase-relatedfamily protein	Cleavage
			**5b**	CGGAUUAUCCCGGCAGCUUCG	3.5	17.04	AT5G23060.1	Ath-Calciumsensing receptor	Translation
			**5c**	UUAGAUCAGGCUGGCAGCUUGU	3.5	17.28	AT5G37020.2	Ath-ARF8	Cleavage
			**5d**	AGAUCAGGCUGGCAGCUUGU	3.5	18.68	AT1G30330.1	Ath-ARF6	Cleavage
							LOC100829021/LOC100837546	Bdi-ARF6/17	
			**5e**	CAGGGCUUGUUGGCAGCUUUA	3.5	19.27	AT3G01820.1	P-loop containingnucleoside triphosphatehydrolases superfamily protein	Cleavage
			**5f**	UUAGAUCAGGCUGGCAGCUUGU	3.5	19.57	AT5G37020.1	Ath-ARF8	Cleavage
6	gma-miR167g, bdi-miR167c,d,ccl-miR167, a,bctr-miR167	UGAAGCUGCCAGCAUGAUCUGA	**6**	UUAGAUCAGGCUGGCAGCUUGU		N/A	LOC100814479	Gma-ARF8	N/A
							LOC100842100	Bdi-ARF12	
							LOC103863707	Bra-ARF8	
							LOC103501352	Cme-ARF8	
7	gma-miR167h	AUCAUGCUGGCAGCUUCAACUGGU	**7**	AGUUGAAGCUGCUGGUGUAAU	3.5	13.31	02G145300	Gma-Methylcrotonoyl-CoAcarboxylase	Cleavage
8	gma-miR167i	UCAUGCUGGCAGCUUCAACUGGU	**8**	UUAGUUGAAGCGGUUAGCGUGA		N/A	LOC100779102	Gma-leucine-richrepeat receptor-likeprotein kinase	N/A
9	ppt-miR167	GGAAGCUGCCAGCAUGAUCCU	**9**	GGCAUCAUGCUGUCAGCUUUC	2.5	N/A	PHYPADRAFT_211955	Ppt-Predictedprotein	Cleavage
10	bna-miR167a,b	UGAAGCUGCCAGCAUGAUCUAA	**10**	UUAGAUCAGGCUGGCAGCUUGU		N/A	AT1G30330.1/AT5G37020.1	Ath-ARF6/8	Cleavage
							LOC103405902	Mdm-ARF8	
							LOC102631382	Csi-ARF8	
11	bna-miR167d	UGAAGCUGCCAGCAUGAUCU	**11**	AGAUCAGGCUGGCAGCUUGU		N/A	AT1G30330.1/AT5G37020.2	Ath-ARF6/8	Cleavage
12	vvi-miR167c	UGAAGCUGCCAGCAUGAUCUC	**12**	GAGAUCAGGCUGGCAGCUUGU	3.5	19.24	LOC100258129	Vvi-ARF	Cleavage
							LOC100242923/LOC100260866	Vvi-ARF6/17	
13	mdm-miR167h–j	UGAAGCUGCCAGCAUGAUCUUA	**13**	GAGAUCAGGCUGGCAGCUUGU		N/A	NM_001294030.1	Mdm-ARF2	N/A
							LOC100301945	Sly-ARF8	
14	mdm-miR167a	AGAUCAUCUGGCAGUUUCACC	**14**	GUGAAAUUGCCGGAUGAUAU		N/A	LOC103428675	Mdm CalcineurinB-like protein 10	N/A

**Abbreviations**: N/A, Not available/could not be identified using psRNATarget tool, alternatively blast used; UmiR167, Unique miR167 sequence; UTS, Unique Target Sequences.
