# Supplementary material for: Coevolution Pattern and Functional Conservation or Divergence of miR167s and their targets across Diverse Plant Species
Source: Sci Rep. 2015 Oct 13;5:14611. doi: 10.1038/srep14611 (PMC4602222; doi:10.1038/srep14611)
Supplement: Supplementary Information [file srep14611-s1.pdf]

Coevolution Pattern and Functional Conservation or Divergence of miR167 and their targets across Diverse Plant Species

Suvakanta Barik, Ashutosh Kumar, Shabari SarkarDas, Sandeep Yadav, Vibhav Gautam, Archita Singh, Sharmila Singh and Ananda K. Sarkar

Supplementary Figure S1. A NJ phylogeny of miR167s using MEGA5. The tree is divided into group I and group II. The group I contains three miR167-3p sequences, whereas all other miRNA167s (-5p) belong to the group II. The scale bar represents the nucleotide substitution rate.

Supplementary Figure S2. Multiple sequence alignment between UTS-2a (target of UmiR167-2), Osa-NBS-LRR (japonica rice cultivar-group) and Osa-NB-ARC (indica rice cultivar-group).

Supplementary Information S3. Novel target sequences and their cleavage or complementarity with miR167s. (**A**) Target sequence of mdm-miR167a and sequenced 5' RLM RACE PCR products (with ligated RNA adapter). The *Mdm-CNBL10* gene sequence has complementary biding sites (yellow) and cleavage sites (where underline starts within mRNAs) and primer location have been indicated. (**B**) Complementarity between UmiR167-7 and *Gma-MCCC* and red arrow shows cleavage site. The *Gma-MCCC* gene sequence has complementary biding sites (yellow) and cleavage sites (where underline starts within mRNAs) and primer location have been indicated. (**C**) Complementarity between UmiR167-8 and *Gma-LRPK* and red arrow shows cleavage site. The *Gma-LRPK* gene sequence has complementary biding sites (yellow) and cleavage sites (where underline starts within mRNAs) and primer location have been indicated

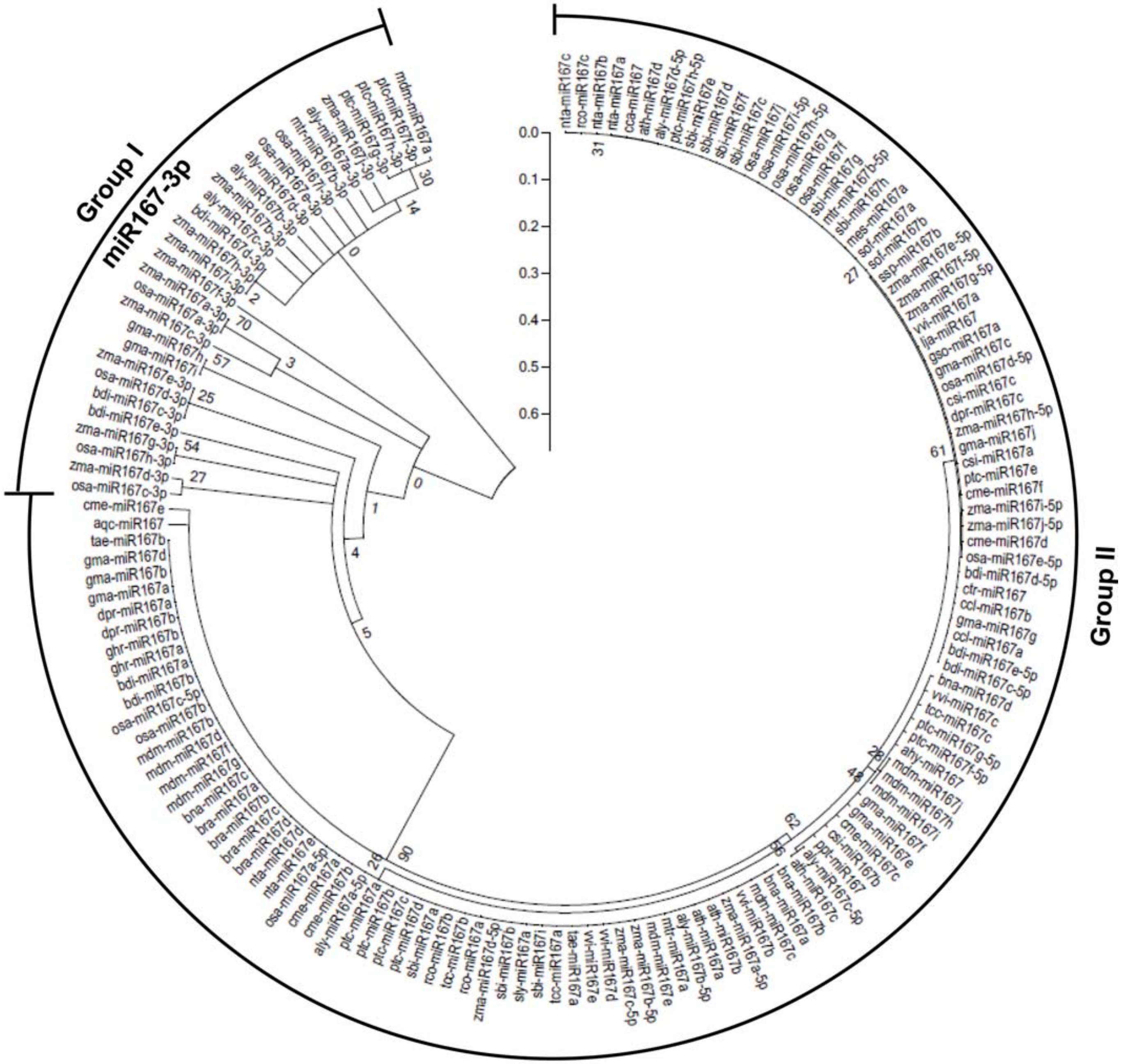

Supplementary Figure S1

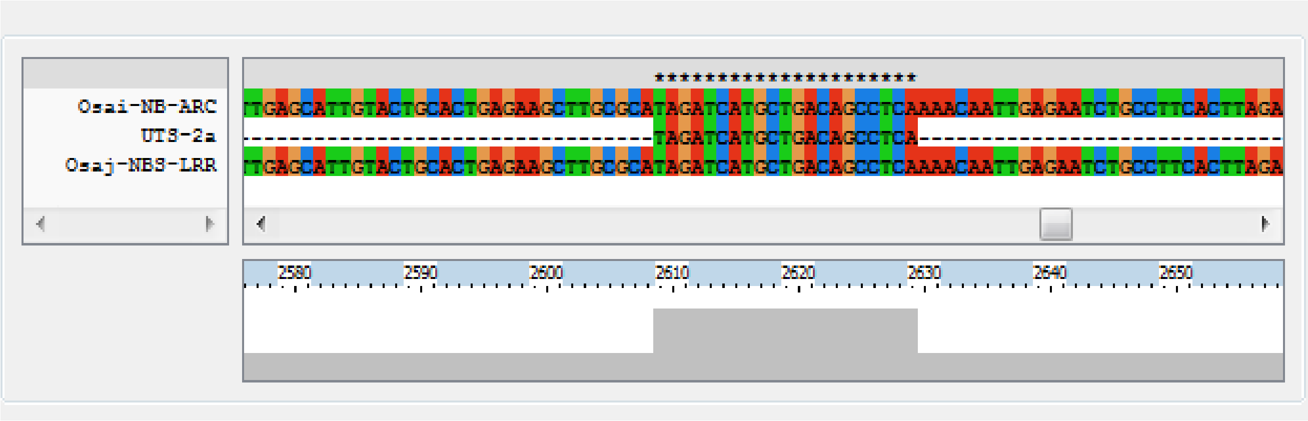

Supplementary Figure S2

Supplementary Information S3

S3A. Target sequence of mdm-miR167a and sequenced 5’ RLM RACE PCR products

***Mdm-CNBL10* sequence**

>gi|657971446|ref|XM_008379288.1| PREDICTED: Malus x domesticacalcineurin B-like protein 10 (LOC103440590), mRNA

5’AGCAAATKTATAATCCGCGCCAGCGGCGCGAGAGAGAAGCAGCCCCTCGAAGCCTTCTGGTGCA
CCCGAGGACATTTTTCATYGAAAAGCACAAGGCACAGAAGAAGTAGCCTCKATCTGATGAATTGT
TGCAGACGACAGCAATGGAATTCTCGAGATCGAGTTCGTTATCAATCGGAGAGAAGATCTGCGCG
GCGTTTATACCGTTTGTAGCTATGGTAGAGGCGCTGGTTTTCGCCGTGAGCGGTTGCTGCAGCCAC
CGTTCCYGCCGCCCAATCAACCGCGGTTTCGCTTGCGGCTATATGTCTCACCTCGCCGATGAAACC
AGATTTACTGTTAATGAGCTGGAGGCGTTGTATGAGCTTTTTAAGAAGCTGAGCAGCTCGATTATC
GACGACGGATCGATTCACAAGGAGGAGCTTCAATTAGCACTATTTCAAACTCCCCAAGGCGAAAA
TCTGTTCCTTGACCGGGTTTTTGATCTATTTGATGAAAAGAAAAATGGTGTCATTGAATTTGATGAG
TTTGTTCATGCACTCAATRTCTTCCATCCTTACGCCCCTATTGATGACAAAATCGATTTTGCATTTC
GGCTGTATGATCTGAGACAAACCGGATTCATTGAGMGAGAAGAGGTTAAGCAAATGGTGATTGCC
ATATTGATGGAATCCGACGTGAAATTGCCGGAT[X] GATATTCTTGAGGAAATCATTGAGAAGACATT
TGCCGACGCTGATGCTGACAAAGATGGGAAAATTAACAAAGAAGAATGGAAGGTTTTCGTGCTTC
GGCATCCATCGCTTCTAAAAAACATGACTCTTCCTTATTTGAAGGACATCACGACAGTATTTCCGA
GTTATATTTTTCACACCGAAGTTGAAGACTGAGCATTTGAAAGCCTTTTCCTACAGAGGATCTTTA
GAAGAAGAGCATAATTCCAAAGCTCCTGCGTATTGCGGAGATACTTCCATCAATTCAAAGGGGGW
MACATCCGAGTTTTAGGCAAGGCCGGGATGGC -3’

(Note: X: cleavage site by mdm-mR167a).

5’ RLM RACE PCR products (from band at ~316 bp)

Sequence 1

>Mdm-CNBL10-Seq1
GAACACGAAAACCTTCCATTCTTCTTTGTTAATTTTCCCATCTTTGTCACATCA
CGTCGGCAAATGTCTTCTCAATGATTTCCTCAAGAATATCTTTCTACTCCTTCA
GTCCATGTCAGTGTCCAC

Sequence 2

>Mdm-CNBL10-Seq2
CGATGGATGCCGAAGCACGAAAACCTTCCATTCTTCTTTGTTAATTTTCCCATC
TTTGTCAGCATCAGCGTCGGCAAATGTCTTCTCAATGATTTCCTCAAGAATATC
TTTCTACTCCTTCAGTCCATGTCAGTGTCC

S3B. Sequence alignment of gma-miR167h and its target *Gma-MCCC*

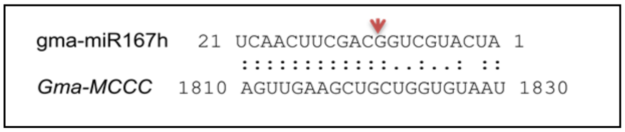

***Gma-MCCC* sequence**

>TC440662 UniRef100_Q42777 Cluster: Methylcrotonoyl-CoA carboxylase subunit alpha, mitochondrial precursor; n=1; Glycine max|Rep: Methylcrotonoyl-CoA carboxylase subunit alpha, mitochondrial precursor - Glycine max (Soybean), complete

GGCACCAACCTCCATGGCTTCTCTGGCGTTGCTCCGCAGAACCACGCTCTCCCAC
TCGCACGTGCGTGCACGAGCCTTCTCTGAGGGGAAGAGCAGCAACAGACACCGC
ATCGAGAAGATTCTAGTAGCCAACCGCGGTGAAATCGCTTGCAGGATCACGAGG
ACCGCGCGGAGGCTCGGCATTCAAACCGTCGCCGTTTACAGCGACGCCGACAAG
GACTCGCTCCACGTTGCGTCCGCCGACAAGGCCATCCGAATCGGACCGCCACCG
GCGCGGCTCAGTTACTTGAACGGAGCCTCCATCGTCGACGCCGCCATTCGCTCCG
GCGCGCAGGCTATTCACCCTGGGTATGGATTCTTGTCGGAGAGTGCTGACTTCGC
CAAGCTCTGTGAGGAGAGTGGTCTCACTTTTATAGGGCCTCCTGCGTCTGCTATT
CGAGATATGGGTGACAAGAGTGCATCGAAGAGAATAATGGGAGCTGCAGGTGTG
CCTCTTGTACCTGGATATCACGGATATGATCAAGATATTGAAAAAATGAAGTTGG
AAGCTGATAGAATTGGATATCCAGTCCTAATTAAGCCAACTCATGGTGGAGGTG
GAAAGGGTATGAGGATTGTACACACTCCAGATGAGTTTGTTGAATCCTTCTTAGC
TGCACAACGGGAAGCAGCTGCTTCTTTTGGCGTCAACACAATATTGTTGGAGAAG
TATATTACACGGCCAAGACACATAGAAGTCCAGATATTTGGGGATAAGCATGGG
AATGTTCTGCATTTGTATGAGCGAGATTGCAGTGTTCAGAGAAGACACCAAAAA
ATAATTGAAGAAGCTCCAGCTCCAAACATTTCTGCTGACTTTCGTGCACAATTGG
GTGTAGCCGCTGTTTCAGCTGCAAAGGCAGTTAATTATTACAATGCTGGGACTGT
GGAGTTTATAGTTGATACAGTCTCTGATGAATTTTACTTTATGGAGATGAATACA
CGTCTTCAGGTTGAGCATCCTGTTACAGAAATGATTGTTGGTCAGGATCTTGTTG
AATGGCAAATCCTTGTTGCCAATGGAGAAGCTCTTCCATTGAGTCAATCACAAGT
ACCCTTATCAGGTCATGCTTTTGAAGCTCGAATCTATGCTGAAAATGTTCAAAAA
GGGTTTCTTCCAGCAACTGGAGTTCTACACCATTATCATGTTCCAGTCTCATCTGC
AGTCCGAGTGGAGACTGGAGTTAAAGAAGGAGACAAAGTTAGCATGCACTATGA
TCCTATGATTGCTAAGCTTGTGGTGTGGGGAGAAAATCGTGCTGCTGCCTTGGTC
AAACTGAAGGATAGTTTGTCAAAGTTTCAGGTTGCAGGTCTGCCAACCAATGTCC
ACTTTCTTCAAAAGCTTGCTAATCACAGGGCATTTGCAATTGGCAATGTGGAGAC
TCATTTTATTGATAACTACAAAGAAGACCTCTTTGTTGATGCTAACAATTCAGTGT
CTGTGAAAGAAGCATATGAAGCTGCTAGACTTAATGCATCCCTGGTGGCCGCAT
GCCTCATTGAAAAAGAGCATTTCATATTGGCTAGAAATCCACCTGGGGGCAGCA
GCTTGCTCCCTATATGGTATTCTTCTCCACCTTTCAGAATCCATCATCAAGCTAAG
CGTAGAATGGAACTTGAGTGGGATAATGAATATGGTAGTGGTAGCTCAAAGATC
ATGAAGCTTACCATCACTTATCAGCCTGATGGGAGATACCTAATTGAGACAGAA
CAAAATGGATCTCCTGTTTTAGAAGTGAAATCAACATATGTAAAAGATAACTATT
TTAGAGTTGAAGCTGCTGGTGTAATCAATGATGTTAATGTAGCTGTTTATTCAAA
GGATCAAATAAGGCATATTCATATTTGGCAAGGGTCCTGTCATCATTATTTCAGA
GAGAAACTAGGCCTGGAGCTGTCTGAGGATGAAGAATCCCAACATAAACCCAAA
GTTGAAACATCAGCCAACCCTCAAGGGACAGTTGTAGCACCCATGGCAGGCTTG
GTAGTTAAGGTTCTAGTGGAGAACAAGACAAGAGTGGAGGAAGGACAACCTGTG
TTAGTATTGGAAGCAATGAAGATGGAGCATGTTGTAAAGCACCTTCTTCTGGCTA
TGTGCATGGGCTTCAACTTATGGTGGGTGAACAGGTTTCAGATGGCAGTGTTCTT

TTCAGTGTAAAGGATCAATAAAATCGCACTTGACAGCTTTACGCCAAGACTTTGG
CAAGGGGGACATTATCTGGAATTCTGGATGGATAGTTATACGCAGGTTAACAAC
CGAACACCCGAACACCTGGGAGTTGATTACTTGCTTAACACTCTTTGTAACTCAA
TTTTTCAAGTTACAAAAGTTGAAATATACTTTACAATAATGCAAGTAGTTCCTCC
AGCCATTAATAACAAAGCTGTAATTTGCTTCATGTTTAGATATTCTTCCATCAACG
GGTCTTCACCTGCCCCTTAAATAAATCTCTAAAGGGGAGATGCCCACACGCCTAA
AATCATGTGATAGTCGGAACACCATATTCCAACTACGAATCCAAGATTCAATTGT
CTTGGGGGAAAC

S3C. Sequence alignment of gma-miR167i and its target *Gma-LRPK*

```
gma-miR167i   20 UCAACUUCGACGGUCGUACU 1
                 :::::::::::::: ::: :
Gma-LRPK     490 AGUUGAAGCUGCCAUCAUCA 509
```

***Gma-LRPK* sequence**

>gi|571475916|ref|XM_003534705.2| PREDICTED: Glycine max uncharacterized LOC100779669 (LOC100779669), transcript variant X1, mRNA
AAATTAATAGTGGGGTCATATCATATTGGATTGTCAAATTATATGTCTATTGCAG
CCTCCTCATGCTGACCATCTCCAATTCCAAACCTTGAATGAAATGCAGAGGCAAA
ATCAGAGGTAAAATTGAGGATGAATAAGTGATGCATCCAAATCAATTAAGCCAA
GTACAGACTTGAAGTTGGGCACTAACTACTGGTAAGGATTTCCCATGTGTGCATT
ATCTACATACCATGGGGTGTTTTTCTTCAAAAGTCATAGCCAGATCAATCAGCTT
CCATGAAGAGAGAAAGAAGAGATCACAGAGAAAAACTAACGGCATTCCATTGCT
GGAAGATCTAATCATCTCCACTGGTGGCAGTGACCAGTACCTTGCTCTTTTCTGT
GCAGCTAACACAGTATCCAACAAACTGCATTCTGGAAGTTTAAGTTCTAACACTT
CCTCAAAACTGGCCATTGAGCCTGCCAGTGCCAGTTCTGAAACTATCAAGAAGTT
GAAGCTGCCATCATCAGCAAGACTAGGCCAAGGAGAAGGGAAACAAATTGAGA
GTGATAAGAACAGGTCCAAAAGTTGGCACCAATTTCCAGAACACATTGTGCAGT
CTCTTGCTCAGGAGAATTCATCTGGTTTTGAAGATAAACATGACTTGAGCTCCAA
GGGTGCTGCGCGTAGTAGGAGCTTTCACACATTGAAGGAATATGATGATATGGTT
AAAAAAATATGGTTGGCCGAATCCCTCACAGATCAGCAAAGTGAGTTTAATGAT
GAAGAAGATGCTGGTTCAGGAGCTAATACAACTCATCATACTGAGGATAGGGAT
TGTGTCATCAAGAAGATGCAACCACCATGCTTAAACAAAAATTATTCATTGGAA
GAAATAGAAGAAAGAAAAGTAGTGAAGGAGACCGACAACTTGAGTACAACATC
AGAAATCAGCACAGTAGCTACAACTCCAAGTCCTAAAAGTCACATATCTAGTTC
AGGTATTAAGAATGAGGGAGTGGTTCCAAGCCATAAAACAAGCACAATTGAGAA
GGGAATTAAGAGAAAAGCTGTGGCAAAGAGGCTAGAATCACTTAGAATCCCAAC
CGGTGTCGAATATCCAGCAATTGCTAGTCTTAGAGAATGGCTCCCTGCAAGTGGA
ATATACTCTCCTGAATCCTATGTCACCCCAAAATTTGGCAGTTATTCTATCATGGA
CATTAGAAATGCAAATGAATCCAGTGAGGATTCTGTATTTAGTCCAGAGTTGGTG
TCTGCCTTTGAACAATGTATGCAAAAACTTGAAGCAGATGAAGAAAACATTCTG
AAACAAATTGTAGAGAATGTGGAGGAAGAAAGTGAAGCAAGCAGCCCCAAGAA
AGAACACCATGCATAGAAAATCAAACAACTGAAGGTGAATTTTGTTAATAATGG
ATAAATCATAGACAAAATTGTTATTTCTACTCCACTACTCTATTTTTCACATCTTC
AATGCCTGTATGCATGTGAATTTTCAGGTCACAATTTTTTTTTTAATTTGAGAGTA
TTCTGAGAAGAGATGCCAATTTACATGGCAGTGAATGTAAATGTACTATGCCTAC
ATCCAATGCCTTGACCACCTTTGTGTATCATATCATAGTTATAAAAAATTTGGAG
AAAACAATTTCGTTGGA
